# Potentially Inappropriate Medication Use in Primary Care in Switzerland

**DOI:** 10.1001/jamanetworkopen.2024.17988

**Published:** 2024-06-21

**Authors:** Simeon Schietzel, Stefan Zechmann, Yael Rachamin, Stefan Neuner-Jehle, Oliver Senn, Thomas Grischott

**Affiliations:** 1 Division of Nephrology and Hypertension, Inselspital, University Hospital of Bern, University of Bern, Bern, Switzerland; 2 Institute of Primary Care, University Hospital Zurich, University of Zurich, Zurich, Switzerland

## Abstract

**Importance:**

Potentially inappropriate medication (PIM) exposes patients to an increased risk of adverse outcomes. Many lists of explicit criteria provide guidance on identifying PIM and recommend alternative prescribing, but the complexity of available lists limits their applicability and the amount of data available on PIM prescribing.

**Objective:**

To determine PIM prevalence and the most frequently prescribed PIMs according to 6 well-known PIM lists and to develop a best practice synthesis for clinicians.

**Design, Setting, and Participants:**

This cross-sectional study used anonymized electronic health record data of Swiss primary care patients aged 65 years or older with drug prescriptions from January 1, 2020, to December 31, 2021, extracted from a large primary care database in Switzerland, the FIRE project. Data analyses took place from October 2022 to September 2023.

**Exposure:**

PIM prescription according to PIM criteria operationalized for use with FIRE data.

**Main Outcomes and Measures:**

The primary outcomes were PIM prevalence (percentage of patients with 1 or more PIMs) and PIM frequency (percentage of prescriptions identified as PIMs) according to the individual PIM lists and a combination of all 6 lists. The PIM lists used were the American 2019 Updated Beers criteria, the French list by Laroche et al, the Norwegian General Practice Norwegian (NORGEP) criteria, the German PRISCUS list, the Austrian list by Mann et al, and the EU(7) consensus list of 7 European countries.

**Results:**

This study included 115 867 patients 65 years or older (mean [SD] age, 76.0 [7.9] years; 55.8% female) with 1 211 227 prescriptions. Among all patients, 86 715 (74.8%) were aged 70 years or older, and 60 670 (52.4%) were aged 75 years or older. PIM prevalence among patients 65 years or older was 31.5% (according to Beers 2019), 15.4% (Laroche), 16.1% (NORGEP), 12.7% (PRISCUS), 31.2% (Mann), 37.1% (EU[7]), and 52.3% (combined list). PIM prevalence increased with age according to every PIM list (eg, according to Beers 2019, from 31.5% at age 65 years or older to 37.4% for those 75 years or older, and when the lists were combined, PIM prevalence increased from 52.3% to 56.7% in those 2 age groups, respectively). PIM frequency was 10.3% (Beers 2019), 3.9% (Laroche), 4.3% (NORGEP), 2.4% (PRISCUS), 6.7% (Mann), 9.7% (EU[7]), and 19.3% (combined list). According to the combined list, the 5 most frequently prescribed PIMs were pantoprazole (9.3% of all PIMs prescribed), ibuprofen (6.9%), diclofenac (6.3%), zolpidem (4.5%), and lorazepam (3.7%). Almost two-thirds (63.5%) of all PIM prescriptions belonged to 5 drug classes: analgesics (26.9% of all PIMs prescribed), proton pump inhibitors (12.1%), benzodiazepines and benzodiazepine-like drugs (11.2%), antidepressants (7.0%), and neuroleptics (6.3%).

**Conclusions and Relevance:**

In this cross-sectional study of adults aged 65 or older, PIM prevalence was high, varied considerably depending on the criteria applied, and increased consistently with age. However, only few drug classes accounted for the majority of all prescriptions that were PIM according to any of the 6 PIM lists, and by considering this manageable number of drug classes, clinicians could essentially comply with all 6 PIM lists. These results raise awareness of the most common PIMs and emphasize the need for careful consideration of their risks and benefits and targeted deprescribing.

## Key Points

**Question:**

What is the prevalence of potentially inappropriate medication (PIM) prescriptions in Swiss primary care according to 6 international PIM lists, and can the majority of potentially inappropriate prescribing be avoided by focusing on a limited set of the most commonly prescribed PIMs?

**Findings:**

In this cross-sectional study of 115 867 patients aged 65 years or older, 52.3% took at least 1 PIM according to any of the 6 PIM lists. Of 1 211 227 prescriptions, 19.3% were PIM, with 63.5% of the latter belonging to the drug classes analgesics, proton pump inhibitors, benzodiazepines, antidepressants, and neuroleptics.

**Meaning:**

The results suggest that the majority of potentially inappropriate prescribing may be avoided by focusing on frequently prescribed PIMs in 5 drug classes.

## Teaser

This cross-sectional study of primary care patients aged 65 years or older in Switzerland assesses the prevalence of potentially inappropriate medication prescriptions (PIMs) according to 6 international PIM lists.

## Introduction

Prescribing of potentially inappropriate medications (PIMs) exposes older adults to increased risk of unfavorable outcomes. PIMs have been associated with hip fractures,^1^ fall-related injuries,^2^ hospitalizations,^1,3,4,5,6,7,8^ increased public health services utilization,^9^ and increased health care expenditure.^10^ A recent meta-analysis found a significant association between the prescription of PIM and cardiovascular events as well as overall mortality.^11^

International lists of explicit criteria provide guidance on PIM identification and alternative prescribing. Widely recognized explicit PIM lists are the American Geriatric Society 2019 updated Beers criteria (Beers 2019),^12^ the French consensus panel list by Laroche et al,^13^ the Norwegian General Practice (NORGEP) criteria,^14^ the German PRISCUS list,^15^ the Austrian consensus panel list by Mann et al,^16^ and the consensus list of 7 European Union countries (EU[7]).^17^

Effective use of existing PIM lists is not straightforward. First, there is considerable variation among different lists in terms of the targeted age group, local drug market, data currency, number of criteria, compilation process, and integration of specific medical conditions or drug-drug interactions. Second, the PIM lists’ application in everyday clinical practice is complicated by the wealth of information provided and the challenges of aligning with local drug markets and prescribing habits. Lastly, awareness of PIM lists as valuable tools for evidence-based risk-benefit analyses is limited.^18^

These challenges in the practical application of PIM lists also reduce the amount of data available on PIM prescribing. Therefore, to expand and consolidate existing data, we aimed to assess PIM prescribing at both the patient and prescription level by applying the aforementioned PIM lists in a large primary care cohort of older adults. Additionally, we aimed to facilitate PIM identification and inform clinical decision-making by presenting the most frequent PIMs and PIM drug classes, along with specific alternative prescribing recommendations as provided by the PIM lists’ authors.

## Methods

### Study Design and Setting

This was a cross-sectional study using data from a large electronic health record–based Swiss primary care database, the FIRE project. Ethical approval and the requirement for informed consent for research using FIRE data has been waived by the competent Ethics Committee of the Canton of Zurich, as it falls outside the scope of the Swiss Federal Act on Research Involving Human Beings (BASEC No. Req-2017-00797). This study followed the Strengthening the Reporting of Observational Studies in Epidemiology (STROBE) reporting guideline.^19^

### FIRE Database and Study Population

The FIRE cohort represents an adult primary care population of central, northern, and eastern Switzerland. Since the FIRE project started in 2009, over 750 individual general practitioners (GPs) (>10% of all Swiss GPs) have voluntarily contributed anonymized clinical routine data from their electronic medical records to the FIRE database, hosted by the Institute of Primary Care of the University Hospital Zurich and the University of Zurich.^20,21^ At the end of 2021, the database held over 12 million consultation records and included nearly 220 000 patients aged 65 years or older (eFigure 1 in Supplement 1). Apart from GP and patient demographics, the database collects reasons for encounters coded according to the *International Classification of Primary Care*, *Revised Second Edition* (*ICPC-2-R*),^22^ drug prescriptions using Anatomical Therapeutic Chemical (ATC) codes^23^ and Global Trade Item Numbers (GTINs),^24^ vital parameters (eg, blood pressure [BP]), clinical data (eg, weight and height), and laboratory test results (eg, estimated glomerular filtration rate [eGFR]).

### Data Query and Preparation

We included patients with a prescription written at any time in 2020 or 2021 when they were at least 65 years of age. For each such prescription (hereinafter called the index prescription), we retrieved its bottom-level ATC code, as well as the patient’s current eGFR (if available), all *ICPC-2-R*–coded reasons for encounters, ATC codes and GTINs of other prescriptions, and BP readings up to the index prescription. We then determined active conditions and comedication at the time of the index prescription based on record dates, package sizes (available from GTINs), and typical daily doses. Finally, we recorded BP as elevated if the systolic BP was greater than 140 mm Hg or the diastolic BP was greater than 90 mm Hg.

### Operationalization of PIM Lists

Two experienced internists (S.S. and S.Z.) independently operationalized all PIM criteria in the 6 PIM lists for the use with FIRE data according to their authors’ explicit or, in the absence of such, presumed intentions. (A seventh well-known list, the STOPP criteria,^25^ was not operationalized because it requires additional clinical knowledge about the patient that is not adequately reflected in the FIRE data.) Difficulties and disagreements were resolved with a third author (S.N.-J.) and the medically trained study statistician (T.G.), who then R coded all operationalized criteria using R, version 4.2.0 (R Project for Statistical Computing).^26^

We performed the following procedure for each PIM list. First, using the WHO ATC/Defined Daily Dose index^23^ and the Swiss Arzneimittel-Kompendium,^27^ each listed substance was assigned all matching ATC codes, and drug classes were then represented by all ATC codes of the individual substances therein, which had been specified by the authors of the original PIM lists for 7 drug classes and compiled in consensus by 3 authors of the present study (S.S., S.Z., and T.G) for another 27 classes.^28^ Second, for each ATC code, we identified all criteria in the PIM list that defined a corresponding prescription to be a PIM, and translated these criteria as closely as possible according to the explicit or assumed intentions of the PIM list’s authors, into conditions on the variables available in FIRE.^28^ Such criteria could apply to dose, duration of use, medical conditions, comedication, BP, and/or kidney function. Dose criteria were operationalized using GTINs; duration of use by the number of prescriptions and the respective GTINs; and medical conditions and comedication by the *ICPC-2-R* and ATC codes active at the time of the index prescription, the substance classes defined in the previous step, and the elevated BP values. Kidney function was operationalized via the current eGFR. Finally, all operationalized criteria were converted into R code^28^ using R, version 4.2.0 (R Project for Statistical Computing),^26^ and applied to the ATC codes of all prescriptions, to determine their PIM status.

### Statistical Analysis

Analyses were conducted from October 2022 to September 2023. We defined PIM prevalence (more precisely, PIM period prevalence for the years 2020 and 2021) as the percentage of patients with at least 1 PIM prescribed between January 1, 2020, and December 31, 2021, and PIM frequency as the percentage of prescriptions (in the same period) identified as PIMs. PIM prevalence was calculated among all included patients as well as in the subsets of patients aged 70 years or older and 75 years or older, according to each individual PIM list and also the combined PIM list (a prescription was considered to be PIM according to the combined PIM list if it qualified as PIM according to at least 1 individual PIM list. PIM frequency was calculated per ATC code, based on all index prescriptions to all included patients, again according to the individual as well as the combined PIM lists. For the most frequent PIMs, we also calculated their percentages on all PIMs prescribed, and we determined the distribution of all prescribed PIMs across important therapeutic drug classes.

Furthermore, we compiled top 10 lists of the most frequently prescribed PIMs according to all individual PIM lists and the combined PIM list. For the top 20 most frequently prescribed PIMs according to the combined PIM list, we compiled their reasons for being PIMs and recommended alternatives from the original publications. Analyses were performed using R, version 4.2.0 (R Project for Statistical Computing).^26^

## Results

### Study Population and Comparison of PIM Lists

Our study included 115 867 primary care patients aged 65 years or older (55.8% female and 44.2% male) with 1 211 227 prescriptions in 2020 or 2021, cared for by 730 GPs from 198 practices. At the time of their first index prescriptions, the patients’ mean (SD) age was 76.0 (7.9) years. Among all patients, 86 715 (74.8%) were aged 70 years or older, and 60 670 (52.4%) were aged 75 years or older. The patients’ analyzed prescriptions dated from a mean (SD) of 5.8 (7.2) different days (IQR, 2-7). General characteristics and the origins^29,30,31,32,33,34,35,36,37,38^ of the 6 PIM lists are summarized in Table 1.

**Table 1.** Characteristics of the 6 PIM Lists

Characteristic	PIM list					
	Beers 2019^12^	Laroche^13^	NORGEP^14^	PRISCUS^15^	Mann^16^	EU(7)^17^
Origin	US	France	Norway	Germany	Austria	Europe
Age group targeted	≥65	≥75	≥70	≥65	≥65^a^	≥65^b^
Experts involved, No.	13	15	144	36	8	30
Sources	Beers 2015^33^; review of new literature	Beers criteria 1991,^34^ 1997,^30^ and 2003^31^; Canadian criteria 1997^32^; adapted Beers criteria 2005^35^; French recommendations 2005^36^	Beers criteria 1991,^34^ 1997,^30^ and 2003^31^; Swedish recommendations^37^; Norwegian study^38^; review of recent literature	Beers 1997^30^ and 2003^31^; Canadian criteria 1997^32^; Laroche 2007^13^; review of recent literature	Beers 1997^30^ and 2003^31^; Canadian criteria 1997^32^; Laroche 2007^13^; PRISCUS 2010^15^; review of recent literature	Beers 1997^30^ and 2003^31^; Canadian criteria 1997^32^; Laroche 2007^13^; PRISCUS 2010^15^; review of recent literature
Substances considered, No.	168	101	32	81	81	73
Drug classes defined, No.	27	6	14	0	0	3
Criteria considered						
Comorbidities	Yes	Yes	No	Yes	No	No
Dose, duration of intake	Yes, yes	Yes, no	Yes, no	Yes, no	Yes, yes	Yes, yes
Drug-drug interactions	Yes	Yes	Yes	No	No	No
Kidney function	Yes	No	No	No	No	No^c^
Other specifics						
Defined list of anticholinergics	Yes	Yes	No	No	No	No
Differentiation: avoid vs caution	Yes	No	No	No	No	NA
Quality of evidence and/or strength of recommendation incorporated	Yes	No	No	No	No	No

Abbreviations: EU(7), consensus list of 7 European Union countries; NORGEP, Norwegian General Practice; PIM, potentially inappropriate medication.

^a^ No specific target age defined. The Austrian PIM list (Mann)^16^ is based on 5 earlier lists with different age cutoffs, as well as on a literature review with an age criterion of 65 years or older, which was then also adopted for the PRISCUS list.^15^

^b^ The EU(7) PIM list is a consensus list from 7 European countries. The study for which the EU(7) PIM list was originally developed^29^ studied people with dementia older than 65 years of age; however, the list is based on a preselection of criteria from Beers 1997^30^ and 2003^31^ (≥65 years), PRISCUS^15^ (≥65 years), Laroche^13^ (≥75 years), and the Canadian criteria^32^ (elderly people, with no age cutoff defined).

^c^ Included as part of the recommendations but not of the actual PIM criteria.

### PIM Prevalence

Among all patients aged 65 years or older, PIM prevalence was 31.5% according to Beers 2019, 15.4% according to Laroche, 16.1% according to NORGEP, 12.7% according to PRISCUS, 31.2% according to Mann, and 37.1% according to EU(7), and it was 52.3% according to the combined PIM list (Table 2). PIM prevalence increased with age according to each individual PIM list. For example, according to Beers 2019, PIM prevalence increased from 31.5% at age 65 years or older to 34.4% at age 70 years or older and to 37.4% at age 75 years or older. According to the combined PIM list, PIM prevalence increased from 52.3% at age 65 years or older to 54.7% at age 70 years or older and to 56.7% at age 75 years or older.

**Table 2.** Age-Dependent PIM Prevalence, Number and Percentage of PIMs per Patient, and PIM Frequency, by PIM List^a^

Outcome	PIM list						
	Beers 2019^12^	Laroche^13^	NORGEP^14^	PRISCUS^15^	Mann^16^	EU(7)^17^	Combined PIM list
**PIM prevalence, by patient age in y, No. (%)**							
≥65	36 475 (31.5)	17 800 (15.4)	18 644 (16.1)	14 702 (12.7)	36 094 (31.2)	43 008 (37.1)	60 639 (52.3)
≥70	29 804 (34.4)	17 800 (20.5)	18 223 (21.0)	11 913 (13.7)	27 421 (31.6)	33 572 (38.7)	47 465 (54.7)
≥75	22 707 (37.4)	17 245 (28.4)	13 472 (22.2)	8998 (14.8)	19 327 (31.9)	24 232 (39.9)	34 405 (56.7)
**PIMs per patient, mean (SD)**							
No.	1.07 (3.02)	0.41 (1.52)	0.45 (1.71)	0.25 (1.00)	0.71 (1.79)	1.02 (2.40)	2.02 (4.29)
%	7.3 (15.6)	3.0 (10.2)	3.2 (10.5)	2.2 (8.9)	7.2 (16.1)	8.3 (16.3)	16.6 (22.9)
**PIM frequency**							
Prescriptions identified as PIMs, No. (%)	124 307 (10.3)	47 683 (3.9)	52 685 (4.3)	28 762 (2.4)	81 703 (6.7)	118 007 (9.7)	234 162 (19.3)

Abbreviations: EU(7), consensus list of 7 European Union countries; NORGEP, Norwegian General Practice; PIM, potentially inappropriate medication.

^a^ Based on 1 211 227 prescriptions to 115 867 primary care patients aged 65 years or older, including 86 715 patients aged 70 years or older, and 60 670 patients aged 75 years or older.

### PIM Number per Patient

The mean (SD) number of PIMs per patient (based on all included patients) ranged from 0.25 (1.00) according to PRISCUS to 1.07 (3.02) according to Beers 2019 (Table 2). According to the combined PIM list, the mean (SD) number of PIMs per patient was 2.02 (4.29) (IQR, 0-2).

### PIM Frequency

Within all 1 211 227 prescriptions analyzed, PIM frequency was 10.3% according to Beers 2019, 3.9% according to Laroche, 4.3% according to NORGEP, 2.4% according to PRISCUS, 6.7% according to Mann, and 9.7% according to EU(7), and the PIM frequency was 19.3% according to the combined PIM list (Table 2).

eFigures 2 and 3 in Supplement 1 provide visual impressions of how PIM prevalence and PIM frequency differ or overlap when different PIM lists are used. The data underlying eFigures 2 and 3 can be found in eTables 1 and 2 in Supplement 1.

### Top 10 Lists

The top 10 most frequently prescribed PIMs according to each individual PIM list and the combined PIM list are shown in Table 3, and full rankings are available online.^28^ The most frequently prescribed PIMs according to the combined PIM list were pantoprazole (9.3% of all PIMs prescribed), ibuprofen (6.9%), diclofenac (6.3%), zolpidem (4.5%), and lorazepam (3.7%). The Figure shows how the top 10 according to the combined PIM list rank among the top 10 of each individual PIM list.

**Table 3.** Absolute Numbers of PIM Prescriptions of the Top 10 Most Frequently Prescribed PIMs and Their Percentages on All PIM Prescriptions, by PIM List^a^

Ranking	PIM prescriptions by PIM list (No. [%])						
	Beers 2019^12^	Laroche^13^	NORGEP^14^	PRISCUS^15^	Mann^16^	EU(7)^17^	Combined PIM list
1	Pantoprazole (14 316 [11.5])	Sodium picosulfate (4332 [9.1])	Diclofenac (4743 [9.0])	Zolpidem (3510 [12.2])	Ibuprofen (16 171 [19.8])	Pantoprazole (21 755 [18.4])	Pantoprazol e (21 755 [9.3])
2	Zolpidem (10 469 [8.4])	Ginkgo folium (4097 [8.6])	Ibuprofen (4632 [8.8])	Nitrofurantoin (3110 [10.8])	Diclofenac (14 800 [18.1])	Diclofenac (14 800 [12.5])	Ibuprofen (16 171 [6.9])
3	Lorazepam (8682 [7.0])	Zolpidem (3842 [8.1])	Chondroitin sulfate (2762 [5.2])	Acemetacin (2864 [10.0])	Lorazepam (8682 [10.6])	Ibuprofen (6646 [5.6])	Diclofenac (14 800 [6.3])
4	Quetiapine (7594 [6.1])	Quetiapine (2593 [5.4])	Oxycodone + naloxone (2638 [5.0])	Trimipramine (2652 [9.2])	Ginkgo folium (5326 [6.5])	Sodium picosulfate (5574 [4.7])	Zolpidem (10 469 [4.5])
5	Pregabalin (4903 [3.9])	Diclofenac (2287 [4.8])	Quetiapine (2547 [4.8])	Liquid paraffin (2011 [7.0])	Tramadol (5168 [6.3])	Ginkgo folium (5326 [4.5])	Lorazepam (8682 [3.7])
6	Oxycodone + Naloxone (3803 [3.1])	Ibuprofen (2225 [4.7])	Lorazepam (2489 [4.7])	Bromazepam (1838 [6.4])	Tramadol + paracetamol (3127 [3.8])	Tramadol (5168 [4.4])	Quetiapine (7601 [3.2])
7	Esomeprazole (3284 [2.6])	Nitrofurantoin (2221 [4.7])	Trimipramine (2221 [4.2])	Etoricoxib (1305 [4.5])	Naproxen + esomeprazole (3087 [3.8])	Esomeprazole (4750 [4.0])	Sodium picosulfate (5574 [2.4])
8	Ibuprofen (2846 [2.3])	Lorazepam (1741 [3.7])	Morphine (1514 [2.9])	Amitriptyline (944 [3.3])	Acemetacin (2864 [3.5])	Loperamide (4300 [3.6])	Ginkgo folium (5326 [2.3])
9	Diclofenac (2834 [2.3])	Trimipramine (1705 [3.6])	Trazodone (1505 [2.9])	Hydroxyzine (891 [3.1])	Pramipexole (2245 [2.7])	Zolpidem (3925 [3.3])	Tramadol (5168 [2.2])
10	Trimipramine (2652 [2.1])	Chondroitin sulfate (1344 [2.8])	Mirtazapine (1491 [2.8])	Doxylamine (811 [2.8])	Liquid paraffin (2011 [2.5])	Tramadol + paracetamol (3127 [2.6])	Pregabalin (4903 [2.1])

Abbreviations: EU(7), consensus list of 7 European Union countries; NORGEP, Norwegian General Practice; PIM, potentially inappropriate medication.

^a^ Based on 1 211 227 prescriptions to 115 867 primary care patients aged 65 years or older.

**Figure.**
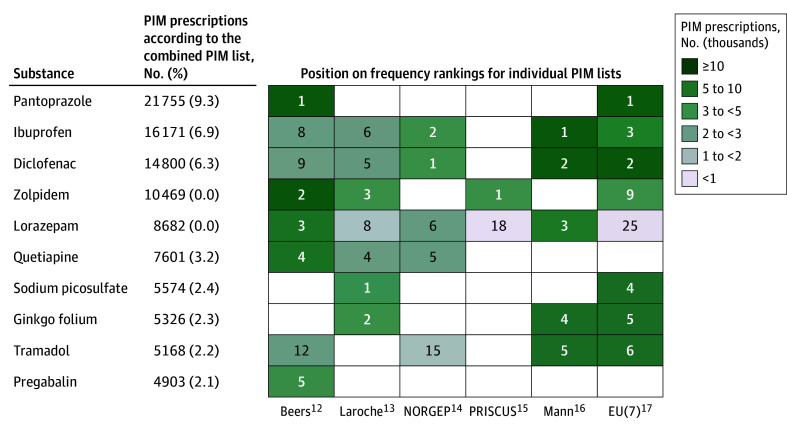
Top 10 Most Frequently Prescribed Potentially Inappropriate Medications (PIMs) According to the Combined PIM List Empty cells indicate that the corresponding PIM list contains no PIM criteria for the substance in question. Beers indicates Beers 2019; EU(7), consensus list of 7 European Union countries; NORGEP, Norwegian General Practice.

The highest fractions of PIM prescriptions according to the combined PIM list fell into the therapeutic drug classes analgesics (26.9% of all PIMs prescribed), proton pump inhibitors (12.1%), benzodiazepines and benzodiazepine-like drugs (11.2%), antidepressants (7.0%), and neuroleptics (6.3%) (Table 4).

**Table 4.** Top 10 Therapeutic Drug Classes With the Most Frequent PIM Prescriptions, According to the Combined PIM List^a^

Ranking of drugs in drug class	PIM prescriptions within drug class, No. (% of total 234 162 PIMs)	PIM lists with mentions of drugs in drug class	Top 10 positions of drugs in drug class, No.^b^
1. Analgesics	63 041 (26.9)	All	21
2. Proton pump inhibitors	28 318 (12.1)	Beers 2019,^12^ EU(7)^17^	4
3. Benzodiazepines and benzodiazepine-like drugs	26 245 (11.2)	All	9
4. Antidepressants	16 301 (7.0)	All	7
5. Neuroleptics	14 712 (6.3)	All	4
6. Antihypertensives	12 424 (5.3)	All	0
7. Laxatives	9555 (4.1)	Beers 2019,^12^ Laroche,^13^ PRISCUS,^15^ Mann,^16^ EU(7)^17^	4
8. Antiepileptics	8162 (3.5)	Beers 2019,^12^ PRISCUS,^15^ Mann,^16^ EU(7)^17^	1
9. Antidementia drugs	5424 (2.3)	Beers 2019,^12^ Laroche,^13^ PRISCUS,^15^ Mann,^16^ EU(7)^17^	3
10. Oral antidiabetics	4633 (2.0)	Beers 2019,^12^ Mann,^16^ EU(7)^17^	0

Abbreviations: EU(7), consensus list of 7 European Union countries; PIM, potentially inappropriate medication

^a^ Based on 1 211 227 prescriptions to 115 867 primary care patients aged 65 years or older.

^b^ From a maximum of 60 top 10 positions according to the 6 individual PIM lists.

Several substances in these classes ranked also among the top 10 most frequently prescribed PIMs according to the individual PIM lists: analgesics held 6 top 10 positions according to Mann (ibuprofen, diclofenac, tramadol, tramadol plus paracetamol, naproxen plus esomeprazole, and acemetacin), 4 according to NORGEP and EU(7), 3 according to Beers 2019 (oxycodone plus naloxone, ibuprofen, diclofenac), and 2 according to Laroche and PRISCUS. Proton pump inhibitors held 2 top 10 positions according to Beers 2019 and EU(7) (pantoprazole and esomeprazole) but are not considered PIM by the other lists. Benzodiazepines and benzodiazepine-like drugs (eg, zolpidem and zopiclone) held 2 top 10 positions according to Beers 2019 and Laroche (zolpidem and lorazepam) and PRISCUS (zolpidem and bromazepam), and 1 according to the other PIM lists.

Analgesics, proton pump inhibitors, and benzodiazepines and benzodiazepine-like drugs together accounted for over half (50.2%) of all PIM prescriptions, the top 5 classes (including antidepressants and neuroleptics) for almost two-thirds (63.5%), and the top 10 classes for more than four-fifths (80.6%) of all PIM prescriptions.

### Recommendations for Alternative Treatments

The original PIM lists contain reasons why substances or substance classes are considered PIMs and recommendations for alternative drugs or therapeutic measures. For the top 20 most frequently prescribed PIMs according to the combined PIM list (Table 3), we compiled these reasons and recommendations in eTable 3 in Supplement 1.

## Discussion

In this cross-sectional study, we applied operationalized criteria from 6 PIM lists to a large sample of older Swiss primary care patients. We found that during the observation period, more than half (52.3%) of the 115 867 older adults included had been prescribed drugs considered PIM by at least 1 of the PIM lists. According to the individual PIM lists, PIM prevalence varied from 12.7% to 37.1%, and it consistently increased with age across all PIM lists considered. PIM frequency (ie, the percentage of prescriptions found to be PIMs), ranged from 2.4% to 10.3% depending on the individual PIM list and reached 19.3% when the criteria from all PIM lists were combined. The majority of PIM prescriptions belonged to only a few drug classes.

Comparing our prevalence estimates to results from other studies is not straightforward, as differences between different PIM concepts and their operationalizations are considerable. However, a recent systematic review^39^ of PIM prevalence in older adults of central and eastern Europe presented similar results, with PIM prevalence within community settings varying between 7% and 41%. The included studies applied a variety of PIM lists (Beers 1997,^30^ Beers 2003,^31^ PRISCUS,^15^ McLeod,^32^ and STOPP/START^25^) individually or in different combinations. A recent global systematic review pooled PIM prevalence estimates among older patients in outpatient services from 94 articles and found 36.7%, a figure within the range of our estimates.^40^

What makes our study unique is the application of 6 different explicit PIM lists and the 6 PIM lists combined to the exact same set of prescriptions for the same population, providing excellent comparability between the different approaches and results from each PIM list. Particularly striking is the different appraisal of proton pump inhibitors (for which only Beers 2019 and EU[7] include PIM criteria), nonsteroidal anti-inflammatory drugs (with top positions on the newer European lists), and substances with low evidence of efficacy (eg, Ginkgo folium) by the French list. The PIM concepts overlap surprisingly poorly, and combining criteria from multiple PIM lists considerably increased the number of prescriptions classified as PIM. Fialová et al^41^ also found a substantial increase of PIM prevalence after combining 3 lists (Beers 1997^30^: 16%, Beers 2003^31^: 25%, McLeod^32^: 32%, and combined: 41%).

Identifying large numbers of PIMs indicates high sensitivity of the criteria applied, which is undoubtedly desirable. However, the criteria’s specificity is no less important. The more specific the characterization of a PIM situation, the more it favors patient-centered medicine rather than serving as a general warning. Also, longer PIM lists do not necessarily identify more PIMs. The number of identified PIMs depends on various factors, such as the number of candidate drugs or drug classes or the complexity and narrowness of the PIM definitions. For instance, PIM prevalence according to the short 36-item NORGEP list (16.1%) is comparable to those of Laroche (15.4%) and PRISCUS (12.7%), 2 rather extensive lists covering 101 and 81 substances, respectively. In this example, the difference lies in the number of drug classes defined as PIM, with 15 in NORGEP compared with only 6 in Laroche and none in PRISCUS.

However, even though a higher number of drug classes in a PIM list may increase sensitivity and thus allow for the identification of more PIM prescriptions, it is not, per se, proof of an unselective approach. The Beers 2019^12^ list covers 168 individual substances and 27 drug classes, compared with only 81 substances and not a single drug class in the Mann list.^16^ Nevertheless, applying the 2 lists to the same prescriptions led to similar PIM prevalence estimates (Beers 2019: 31.5%, Mann: 31.2%). This can be explained, at least partly, by the high number of precisely formulated criteria in the Beers 2019^12^ list, which reduces sensitivity and thus the number of PIMs identified.

As different as the 6 PIM lists (Table 1) and the resulting PIM prevalences and frequencies were (Table 2), the following similarities emerged: first, PIM prevalence was high, exceeding 10% regardless of the PIM list used (and even exceeding 30% for 3 lists). Second, PIM prevalence increased with age according to all 6 PIM lists. Third, analgesics and benzodiazepines and benzodiazepine-like drugs accounted for over a third of all PIM prescriptions, being the only 2 drug classes with substances in the top 10 of every list, holding 21 and 9 out of the 60 top 10 positions, respectively. Last, the majority of PIMs (63.5%) belonged to only 5 drug classes. Therefore, in everyday clinical practice, only a few drug classes need to be kept in mind to be aware of the most common PIMs, with analgesics and benzodiazepines and benzodiazepine-like drugs posing the greatest risk of inappropriate prescribing. In a systematic review of 36 PIM studies including older Beers criteria, the Laroche and NORGEP lists and other concepts, Motter et al also found benzodiazepines and nonsteroidal anti-inflammatory drugs to be the most frequently prescribed PIM classes.^42^

As the population ages, the number of patients with conditions that make certain prescriptions potentially inappropriate will increase. Thus, PIM lists have great potential to become an increasingly valuable tool to foster appropriate prescribing while respecting patients’ needs and preferences. By their very nature, PIM lists advocate cautious prescribing, but it is crucial not to forget the reasons why a PIM was considered in the first place. It is the responsibility of the prescribing physician to balance needs against potential risks so that PIM lists do not lead to overly restrictive prescribing.

Our presentation of the 20 most frequently prescribed PIMs with prescribing alternatives from all 6 PIM lists (eTable 3 in Supplement 1) represents the synthesis of our analyses of the 6 original PIM lists into a single, easy-to-navigate guide for practical use, and we invite clinically interested readers in particular to explore it, in the hope that it will help to increase awareness of the most common PIMs, facilitate their appropriate deprescribing based on individual risk-benefit considerations, and ultimately improve medication safety and quality of care patients aged 65 years or older.

### Strengths and Limitations

Our study has several strengths. To our knowledge, we are the first to apply 6 internationally recognized PIM lists to the same population, allowing for comparative analysis. Furthermore, we have not found other studies featuring a comparable level of detail regarding the operationalization of explicit PIM criteria across multiple lists from 2 continents. We presented PIM prevalences by age group, which allows the results to be interpreted in the light of current demographic challenges. Finally, our top 10 and top 20 lists, along with the rationales and recommendations provided by the authors of the original PIM lists, have great potential to facilitate identification and management of PIMs in clinical practice, and thereby contribute to improved patient care and safety.

Our study also has limitations. Although the FIRE database allows analyses of a large primary care population, results cannot be generalized to other settings (eg, hospitalized patients, nursing homes) or drug markets (eg, different countries). In addition, all operationalizations of PIM lists generally involve some degree of subjectivity. However, we paid utmost attention to every detail and appreciated the explicit and presumed intentions of the PIM lists’ authors to maximize our results’ validity.

## Conclusions

In this cross-sectional study, PIM prevalence varied greatly depending on the PIM list used but was high and further increased with age according to all lists considered. Newer PIM lists showed a tendency to relax completeness and rigor for less complexity. Analgesics, benzodiazepines and benzodiazepine-like drugs, antidepressants, and neuroleptics were major PIM drug classes according to each PIM list, narrowing the spectrum of PIM considerations in everyday clinical practice, while highly detailed PIM lists with appropriate alternatives may guide a more nuanced approach when needed.

## Notes

Supplement 1. **eFigure 1.** FIRE database/study flowchart. **eFigure 2.** Patients with PIMs according to different PIM lists: Approximately area proportional Venn (Euler) diagram showing the number of 115 867 primary care patients ≥65 years with at least one PIM prescription according to one or multiple PIM lists **eTable 1.** Data underlying eFigure 2 **eFigure 3.** PIM prescriptions according to different PIM lists: approximately area proportional Venn (Euler) diagram showing the numbers of a total of 1 211 227 prescriptions to 115 867 primary care patients ≥65 years identified as PIMs according to one or multiple PIM lists **eTable 2.** Data underlying eFigure 3 **eTable 3.** Top 20 most frequently prescribed PIMs according to the combined PIM list, together with the original PIM lists’ authors’ rationales and recommendations for alternative prescribing

Supplement 2.
Data Sharing Statement
