# Supplementary material for: Potentially Inappropriate Medication Use in Primary Care in Switzerland
Source: JAMA Netw Open. 2024 Jun 21;7(6):e2417988. doi: 10.1001/jamanetworkopen.2024.17988 (PMC11193127; doi:10.1001/jamanetworkopen.2024.17988)
Supplement: Supplement 1. — eFigure 1. FIRE database/study flowchart. eFigure 2. Patients with PIMs according to different PIM lists: Approximately area proportional Venn (Euler) diagram showing the number of 115 867 primary care patients ≥65 years with at least one PIM prescription according to one or multiple PIM lists eTable 1. Data underlying eFigure 2 eFigure 3. PIM prescriptions according to different PIM lists: approximately area proportional Venn (Euler) diagram showing the numbers of a total of 1 211 227 prescriptions to 115 867 primary care patients ≥65 years identified as PIMs according to one or multiple PIM lists eTable 2. Data underlying eFigure 3 eTable 3. Top 20 most frequently prescribed PIMs according to the combined PIM list, together with the original PIM lists’ authors’ rationales and recommendations for alternative prescribing [file jamanetwopen-e2417988-s001.pdf]

Supplemental Online Content

Schietzel S, Zechmann S, Rachamin Y, Neuner-Jehle, Senn O, Grischott T. Potentially inappropriate medication use in primary care in Switzerland. *JAMA Netw Open*. 2024;7(6):e2417988.
doi:10.1001/jamanetworkopen.2024.17988

eFigure 1. FIRE database/study flowchart.

eFigure 2. Patients with PIMs according to different PIM lists: Approximately area proportional Venn (Euler) diagram showing the number of 115,867 primary care patients ≥65 years with at least one PIM prescription according to one or multiple PIM lists.

eTable 1. Data underlying eFigure 2.

eFigure 3. PIM prescriptions according to different PIM lists: approximately area proportional Venn (Euler) diagram showing the numbers of a total of 1,211,227 prescriptions to 115,867 primary care patients ≥65 years identified as PIMs according to one or multiple PIM lists.

eTable 2. Data underlying eFigure 3.

eTable 3. Top 20 most frequently prescribed PIMs according to the combined PIM list, together with the original PIM lists' authors' rationales and recommendations for alternative prescribing.

This supplemental material has been provided by the authors to give readers additional information about their work.

© 2024 Schietzel S. *JAMA Network Open.*

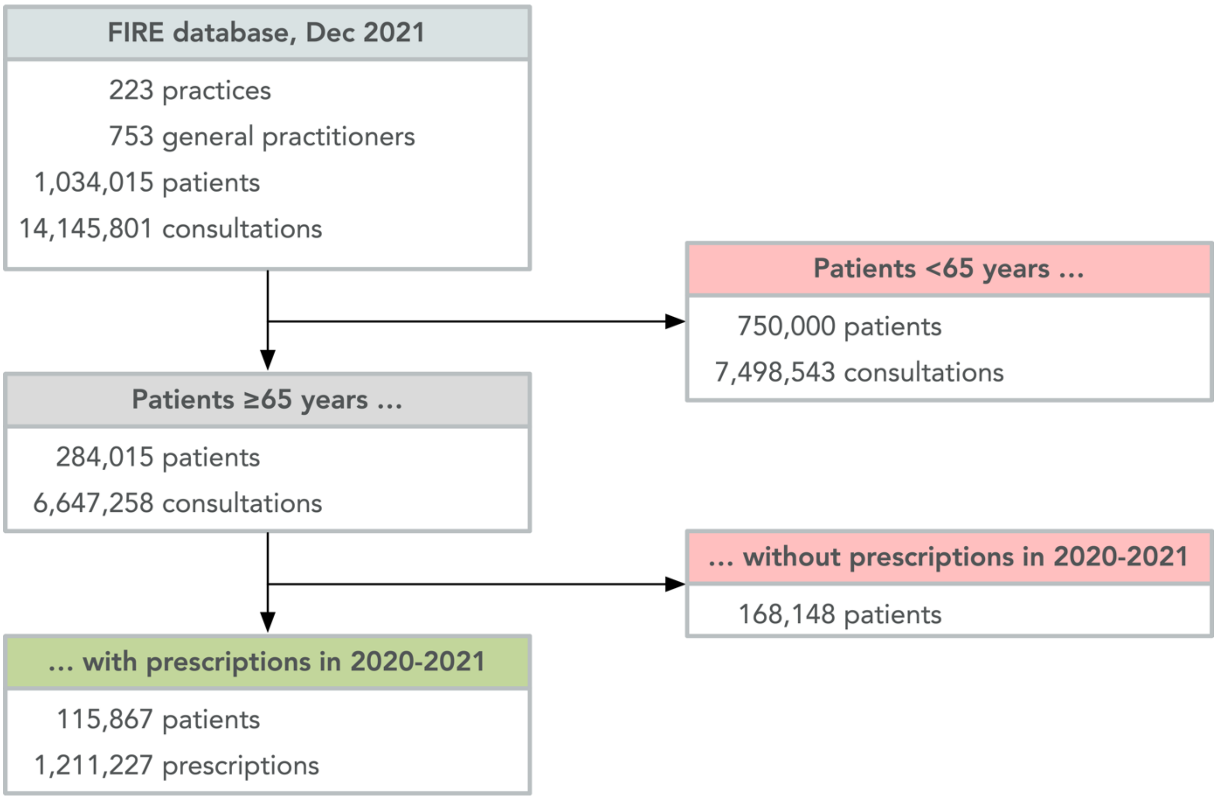

eFigure 1. FIRE database/study flowchart.

© 2024 Schietzel S. *JAMA Network Open.*

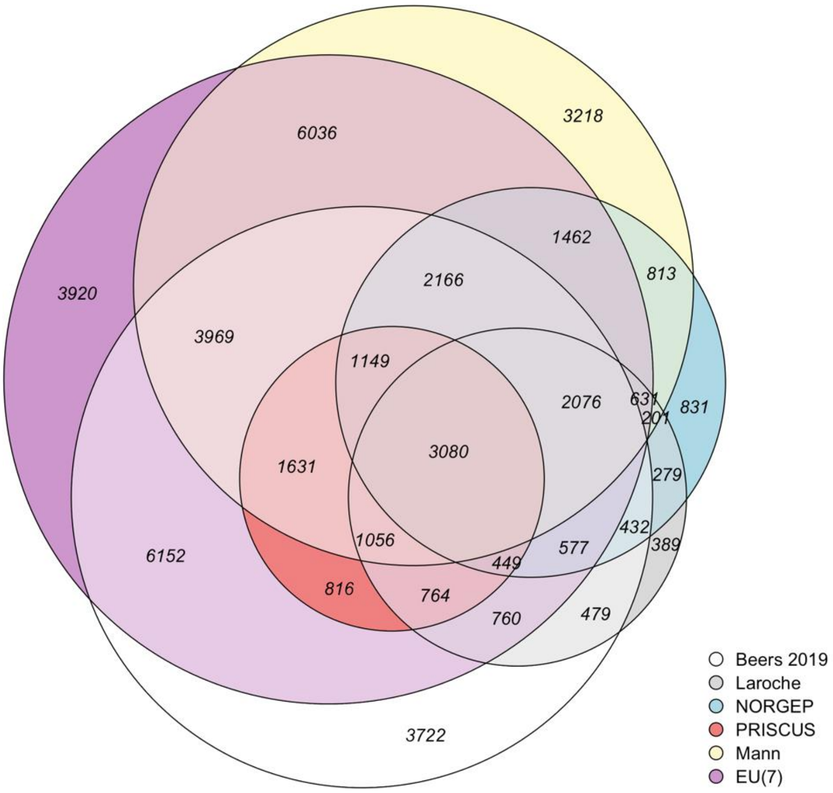

eFigure 2. Patients with PIMs according to different PIM lists: Approximately area proportional Venn (Euler) diagram showing the number of 115,867 primary care patients ≥65 years with at least one PIM prescription according to one or multiple PIM lists.

eTable 1. Data underlying eFigure 2.

**Intersecting PIM lists**	**No. of patients with PIMs**			
	original	fitted	residuals	regionError
Beers 2019	3722	3692.1	29.9	0.02
Laroche	389	268.224	120.776	0
NORGEP	831	753.559	77.441	0.003
PRISCUS	455	0	455	0.008
Mann	3218	3246.049	-28.049	0.019
EU(7)	3920	3953.392	-33.392	0.023
Beers 2019 & Laroche	479	815.472	-336.472	0.01
Beers 2019 & NORGEP	765	0	765	0.013
Beers 2019 & PRISCUS	270	0	270	0.004
Beers 2019 & Mann	1332	0	1332	0.022
Beers 2019 & EU(7)	6152	6130.393	21.607	0.034
Laroche & NORGEP	279	320.295	-41.295	0.002
Laroche & PRISCUS	404	0	404	0.007
Laroche & Mann	254	0	254	0.004
Laroche & EU(7)	786	0	786	0.013
NORGEP & PRISCUS	5	0	5	0
NORGEP & Mann	813	801.809	11.191	0.004
NORGEP & EU(7)	215	0	215	0.004
PRISCUS & Mann	608	0	608	0.01
PRISCUS & EU(7)	286	0	286	0.005
Mann & EU(7)	6036	5946.359	89.641	0.032

© 2024 Schietzel S. *JAMA Network Open.*

Beers 2019 & Laroche & NORGEP	432	258.077	173.923	0.001
Beers 2019 & Laroche & PRISCUS	229	0	229	0.004
Beers 2019 & Laroche & Mann	188	0	188	0.003
Beers 2019 & Laroche & EU(7)	760	759.074	0.926	0.004
Beers 2019 & NORGEP & PRISCUS	100	0	100	0.002
Beers 2019 & NORGEP & Mann	524	0	524	0.009
Beers 2019 & NORGEP & EU(7)	601	0	601	0.01
Beers 2019 & PRISCUS & Mann	434	0	434	0.007
Beers 2019 & PRISCUS & EU(7)	816	822.889	-6.889	0.005
Beers 2019 & Mann & EU(7)	3969	4077.613	-108.613	0.025
Laroche & NORGEP & PRISCUS	16	0	16	0
Laroche & NORGEP & Mann	201	28.064	172.936	0.003
Laroche & NORGEP & EU(7)	116	0.041	115.959	0.002
Laroche & PRISCUS & Mann	82	0	82	0.001
Laroche & PRISCUS & EU(7)	148	0	148	0.002
Laroche & Mann & EU(7)	1330	0	1330	0.022
NORGEP & PRISCUS & Mann	183	0	183	0.003
NORGEP & PRISCUS & EU(7)	67	0	67	0.001
NORGEP & Mann & EU(7)	1462	1728.435	-266.435	0.014
PRISCUS & Mann & EU(7)	557	0	557	0.009
Beers 2019 & Laroche & NORGEP & PRISCUS	307	0	307	0.005
Beers 2019 & Laroche & NORGEP & Mann	463	0	463	0.008
Beers 2019 & Laroche & NORGEP & EU(7)	577	631.111	-54.111	0.004
Beers 2019 & Laroche & PRISCUS & Mann	139	0	139	0.002
Beers 2019 & Laroche & PRISCUS & EU(7)	764	645.033	118.967	0.002
Beers 2019 & Laroche & Mann & EU(7)	1255	0	1255	0.021
Beers 2019 & NORGEP & PRISCUS & Mann	147	0	147	0.002
Beers 2019 & NORGEP & PRISCUS & EU(7)	177	0	177	0.003
Beers 2019 & NORGEP & Mann & EU(7)	2166	2060.738	105.262	0.01
Beers 2019 & PRISCUS & Mann & EU(7)	1631	1814.36	-183.36	0.013
Laroche & NORGEP & PRISCUS & Mann	92	0	92	0.002
Laroche & NORGEP & PRISCUS & EU(7)	33	0	33	0.001
Laroche & NORGEP & Mann & EU(7)	631	79.672	551.328	0.009
Laroche & PRISCUS & Mann & EU(7)	331	0	331	0.005
NORGEP & PRISCUS & Mann & EU(7)	233	0	233	0.004
Beers 2019 & Laroche & NORGEP & PRISCUS & Mann	275	0	275	0.005
Beers 2019 & Laroche & NORGEP & PRISCUS & EU(7)	449	116.809	332.191	0.005
Beers 2019 & Laroche & NORGEP & Mann & EU(7)	2076	1975.572	100.428	0.009
Beers 2019 & Laroche & PRISCUS & Mann & EU(7)	1056	436.533	619.467	0.008
Beers 2019 & NORGEP & PRISCUS & Mann & EU(7)	1149	712.31	436.69	0.003
Laroche & NORGEP & PRISCUS & Mann & EU(7)	179	0	179	0.003
Beers 2019 & Laroche & NORGEP & PRISCUS & Mann & EU(7)	3080	3172.956	-92.956	0.019

© 2024 Schietzel S. *JAMA Network Open.*

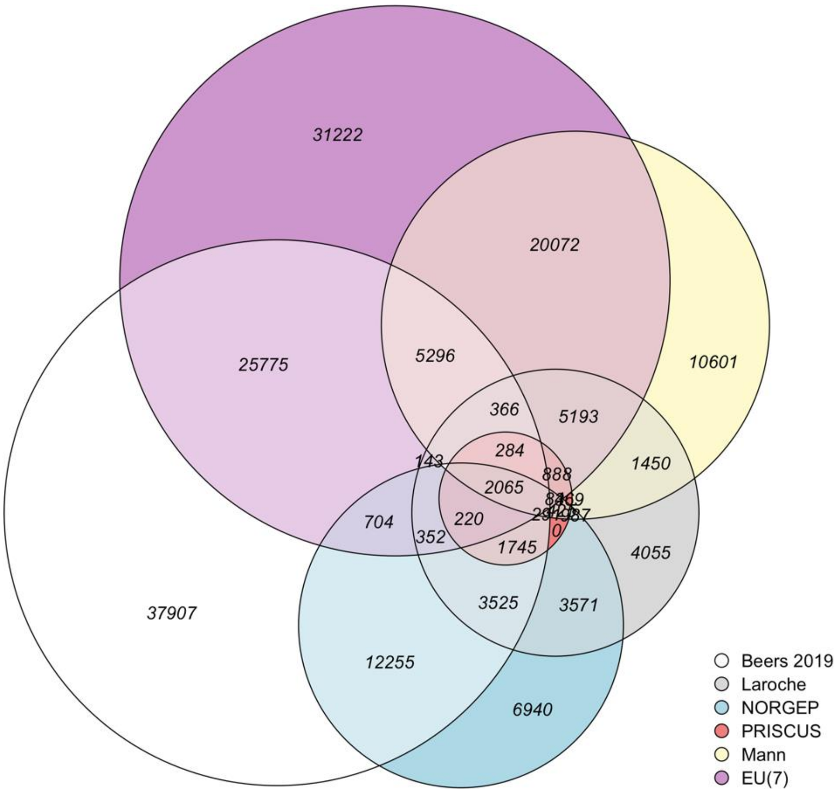

eFigure 3. PIM prescriptions according to different PIM lists: Approximately area proportional Venn (Euler) diagram showing the numbers of a total of 1,211,227 prescriptions to 115,867 primary care patients ≥65 years identified as PIMs according to one or multiple PIM lists.

eTable 2. Data underlying eFigure 3.

**Intersecting PIM lists**	**No. of PIM prescriptions**			
	original	fitted	residuals	regionError
Beers 2019	37907	38041.433	-134.433	0.053
Laroche	4055	3715.99	339.01	0.004
NORGEP	6940	7058.342	-118.342	0.010
PRISCUS	1884	0	1884	0.008
Mann	10601	10317.478	283.522	0.013
EU(7)	31222	31275.718	-53.718	0.043
Beers 2019 & Laroche	4044	0	4044	0.017
Beers 2019 & NORGEP	12255	11877.856	377.144	0.015
Beers 2019 & PRISCUS	1106	0	1106	0.005
Beers 2019 & Mann	7423	0	7423	0.032
Beers 2019 & EU(7)	25775	25403.447	371.553	0.033
Laroche & NORGEP	3571	3904.869	-333.869	0.007
Laroche & PRISCUS	1707	0	1707	0.007
Laroche & Mann	1450	3113.478	-1663.478	0.011
Laroche & EU(7)	6234	0	6234	0.027
NORGEP & PRISCUS	0	0	0	0
NORGEP & Mann	2888	0	2888	0.012
NORGEP & EU(7)	27	0	27	0
PRISCUS & Mann	1944	0	1944	0.008
PRISCUS & EU(7)	1201	0	1201	0.005
Mann & EU(7)	20072	20311.707	-239.707	0.029

© 2024 Schietzel S. *JAMA Network Open.*

Beers 2019 & Laroche & NORGEP	3525	3040.205	484.795	0.002
Beers 2019 & Laroche & PRISCUS	1309	0	1309	0.006
Beers 2019 & Laroche & Mann	760	0	760	0.003
Beers 2019 & Laroche & EU(7)	143	72.509	70.491	0
Beers 2019 & NORGEP & PRISCUS	551	0	551	0.002
Beers 2019 & NORGEP & Mann	2498	0	2498	0.011
Beers 2019 & NORGEP & EU(7)	704	2092.328	-1388.328	0.009
Beers 2019 & PRISCUS & Mann	2722	0	2722	0.012
Beers 2019 & PRISCUS & EU(7)	2333	0	2333	0.010
Beers 2019 & Mann & EU(7)	5296	5163.278	132.722	0.006
Laroche & NORGEP & PRISCUS	0	145.566	-145.566	0.001
Laroche & NORGEP & Mann	987	38.119	948.881	0.004
Laroche & NORGEP & EU(7)	49	0	49	0
Laroche & PRISCUS & Mann	369	27.767	341.233	0.001
Laroche & PRISCUS & EU(7)	566	0	566	0.002
Laroche & Mann & EU(7)	5193	3494.079	1698.921	0.002
NORGEP & PRISCUS & Mann	804	0	804	0.003
NORGEP & PRISCUS & EU(7)	222	0	222	0.001
NORGEP & Mann & EU(7)	3708	0	3708	0.016
PRISCUS & Mann & EU(7)	943	0	943	0.004
Beers 2019 & Laroche & NORGEP & PRISCUS	1745	983.004	761.996	0.002
Beers 2019 & Laroche & NORGEP & Mann	2010	0	2010	0.009
Beers 2019 & Laroche & NORGEP & EU(7)	352	1057.578	-705.578	0.004
Beers 2019 & Laroche & PRISCUS & Mann	238	0	238	0.001
Beers 2019 & Laroche & PRISCUS & EU(7)	2510	0	2510	0.011
Beers 2019 & Laroche & Mann & EU(7)	366	1840.582	-1474.582	0.009
Beers 2019 & NORGEP & PRISCUS & Mann	146	0	146	0.001
Beers 2019 & NORGEP & PRISCUS & EU(7)	191	0	191	0.001
Beers 2019 & NORGEP & Mann & EU(7)	3213	0	3213	0.014
Beers 2019 & PRISCUS & Mann & EU(7)	932	0	932	0.004
Laroche & NORGEP & PRISCUS & Mann	425	127.337	297.663	0.001
Laroche & NORGEP & PRISCUS & EU(7)	111	0	111	0
Laroche & NORGEP & Mann & EU(7)	1390	0	1390	0.006
Laroche & PRISCUS & Mann & EU(7)	888	337.242	550.758	0.002
NORGEP & PRISCUS & Mann & EU(7)	320	0	320	0.001
Beers 2019 & Laroche & NORGEP & PRISCUS & Mann	291	22.551	268.449	0.001
Beers 2019 & Laroche & NORGEP & PRISCUS & EU(7)	220	1474.826	-1254.826	0.007
Beers 2019 & Laroche & NORGEP & Mann & EU(7)	742	10.415	731.585	0.003
Beers 2019 & Laroche & PRISCUS & Mann & EU(7)	284	1178.803	-894.803	0.005
Beers 2019 & NORGEP & PRISCUS & Mann & EU(7)	651	0	651	0.003
Laroche & NORGEP & PRISCUS & Mann & EU(7)	84	50.965	33.035	0
Beers 2019 & Laroche & NORGEP & PRISCUS & Mann & EU(7)	2065	1207.164	857.836	0.002

© 2024 Schietzel S. *JAMA Network Open.*

eTable 3. Top 20 most frequently prescribed PIMs according to the combined PIM list, together with the original PIM lists' [2-7] authors' rationales and recommendations for alternative prescribing.

**1. Pantoprazole**	
**Why PIM**	**Beers 2019**: Proton-pump inhibitors: "Risk of Clostridium difficile infection and bone loss and fractures."<br>**EU(7)**: PPI (>8 weeks): "Long-term high dose PPI therapy is associated with an increased risk of C. difficile infection and hip fracture. Inappropriate if used >8 weeks in maximal dose without clear indication."
**Suggestion**	**Beers 2019**: "Avoid scheduled use for >8 weeks unless for high-risk patients (eg, oral corticosteroids or chronic NSAID use), erosive esophagitis, Barrett esophagitis, pathological hypersecretory condition, or demonstrated need for maintenance treatment (eg, because of failure of drug discontinuation trial or H2-receptor antagonists)."<br>**EU(7)**: No alternatives suggested.
**2. Ibuprofen**	
**Why PIM**	**Beers 2019**: Non–cyclooxygenase-selective NSAIDs, oral: "Increased risk of GI bleeding or peptic ulcer disease in high-risk groups, including those >75 years or taking oral or parenteral corticosteroids, anticoagulants, or antiplatelet agents; use of proton-pump inhibitor or misoprostol reduces but does not eliminate risk. Upper gastrointestinal ulcers, gross bleeding, or perforation caused by NSAIDs occur in ~1% of patients treated for 3-6 months and in ~2%-4% of patients treated for 1 year; these trends continue with longer duration of use. Also can increase blood pressure and induce kidney injury. Risks are dose related." Heart failure: "Potential to promote fluid retention and/or exacerbate heart failure." History of gastric or duodenal ulcers: "May exacerbate existing ulcers or cause new/additional ulcers." Chronic kidney disease stage 4 or higher (creatinine clearance <30 mL/min): "May increase risk of acute kidney injury and further decline of renal function."<br>**Laroche**: Concomitant use of 2 or more NSAIDs: "No enhancement of efficacy, albeit increase of adverse effect risk."<br>**NORGEP**: NSAID (or coxib) + ACE inhibitor (or ARB): "NSAID combinations: Increased risk of renal failure." NSAID + diuretic: "Reduced effect of diuretics." NSAID + glucocorticoid: "Increased risk of intestinal bleeding. Risk of fluid retention." NSAID + SSRI: "Increased risk of gastrointestinal bleeding." Warfarin + NSAID: "Warfarin combinations: Increased risk of intestinal bleeding."<br>**Mann**: Anti-inflammatory drugs: "Serious adverse drug reactions: gastrointestinal ulcers, bleeding, kidney and liver insufficiency, hypertension."<br>**EU(7)**: Ibuprofen (>3×400 mg/d or for a period longer than one week): "Risk of GI bleeding and increased risk of cardiovascular complications at higher doses (>1200 mg/d), especially in case of previous cardiovascular disease."
**Suggestion**	**Beers 2019**: "Avoid chronic use, unless other alternatives are not effective and patient can take gastroprotective agent (proton-pump inhibitor or misoprostol)." Heart failure: "Avoid, or use with caution." History of gastric or duodenal ulcers: "Avoid unless other alternatives are not effective and patient can take gastroprotective agent (ie, proton-pump inhibitor or misoprostol)." Chronic kidney disease stage 4 or higher (creatinine clearance <30 mL/min): "Avoid."<br>**Laroche**: "Use only one NSAID."<br>**NORGEP**: No recommendations given.<br>**Mann**: "In the analgetic indication: Paracetamol, metamizole, hydromorphone."<br>**EU(7)**: "The risk of bleeding may be reduced if combined with proton-pump inhibitors (use <8 weeks, low dose).
**3. Diclofenac**	
**Why PIM**	Refer to ibuprofen, except:<br>**EU(7)**: "Very high risk of GI bleeding, ulceration, or perforation, which may be fatal; cardiovascular contraindications."
**Suggestion**	Refer to ibuprofen, except:<br>**EU(7)**: Dose adjustment: "50 mg/d; start using low dose; the risk of bleeding may be reduced if combined with proton-pump inhibitors (use <8 weeks, low dose)." Alternative

© 2024 Schietzel S. *JAMA Network Open.*

	drugs: "Paracetamol; ibuprofen (≤3 × 400 mg/d or for a period shorter than one week); naproxen."
**4. Zolpidem**	
**Why PIM**	**Beers 2019**: Nonbenzodiazepine, benzodiazepine receptor agonist hypnotics (ie, "Z-drugs"): "Nonbenzodiazepine benzodiazepine receptor agonist hypnotics (ie, Z drugs) have adverse events similar to those of benzodiazepines in older adults (eg, delirium, falls, fractures); increased emergency room visits/hospitalizations; motor vehicle crashes; minimal improvement in sleep latency and duration." Delirium: "Avoid in older adults with or at high risk of delirium because of potential of inducing or worsening delirium." Dementia or cognitive impairment: "Avoid because of adverse CNS effects." History of falls or fractures: "May cause ataxia, impaired psychomotor function, syncope, additional falls." Drug-drug interactions; any combination of three or more CNS-active drugs: "Increased risk of falls and of fracture."<br>**Laroche**: >5 mg/d: "No proven improvement of efficacy when the daily dose is above half that prescribed to young adults and increase of adverse effects."<br>**PRISCUS**: >5 mg/d: "Risk of falling and hip fracture; delayed reaction time; psychiatric reactions (sometimes paradoxical, e.g., agitation, irritability, hallucinations, psychosis); cognitive impairment."<br>**EU(7)**: >5 mg/d: "Risk of falls and hip fracture, prolonged reaction time, psychiatric reactions (which can be paradoxical, e.g. agitation, irritability, hallucinations, psychosis), cognitive impairment and depression."
**Suggestion**	**Beers 2019**: Nonbenzodiazepine, benzodiazepine receptor agonist hypnotics (ie, "Z-drugs"): "Avoid." Delirium: "Avoid." Dementia or cognitive impairment: "Avoid." History of falls or fractures: "Avoid unless safer alternatives are not available. If the drug must be used, consider reducing use of other CNS-active medications that increase risk of falls and fractures (ie, antiepileptics, opioid-receptor agonists, antipsychotics, antidepressants, nonbenzodiazepine and benzodiazepine receptor agonist hypnotics, other sedatives/hypnotics) and implement other strategies to reduce fall risk." Drug-drug interactions; any combination of three or more CNS-active drugs: "Avoid total of three or more CNS-active drugs; minimize number of CNS-active drugs."<br>**Laroche**: "Dose of short- or intermediate- half-life benzodiazepine ≤ half the dose given in young subjects."<br>**PRISCUS**: Alternatives: "Valerian; sedating antidepressants (trazodone, mianserin, mirtazapine); zolpidem (≤5 mg/d); opipramol; low-potency neuroleptic drugs (melperone, pipamperone); non-pharmacological treatment of sleep disturbances (sleep hygiene)." Precautions: "Clinical monitoring for adverse effects (cognitive function, vigilance, regular fall history, testing of gait steadiness, psychopathology, ataxia); dosing recommendation: lowest possible dose, up to half of the usual dose, taper in and out, shortest possible duration of treatment."<br>**EU(7)**: Dose adjustment: "Use the lowest possible dose, up to half of the usual dose, taper in and out, shortest possible duration of treatment."
**5. Lorazepam**	
**Why PIM**	**Beers 2019**: Short and intermediate acting benzodiazepines: "Older adults have increased sensitivity to benzodiazepines and decreased metabolism of long- acting agents; in general, all benzodiazepines increase risk of cognitive impairment, delirium, falls, fractures, and motor vehicle crashes in older adults." Delirium: "Avoid in older adults with or at high risk of delirium because of potential of inducing or worsening delirium." Dementia or cognitive impairment: "Avoid because of adverse CNS effects." History of falls or fractures: "May cause ataxia, impaired psychomotor function, syncope, additional falls; shorter- acting benzodiazepines are not safer than long-acting ones." Drug-drug interactions; any combination of three or more CNS-active drugs: "Increased risk of falls and of fracture."<br>**Laroche**: >3 mg/d: "No proven improvement of efficacy when the daily dose is above half that prescribed to young adults and increase of adverse effects."<br>**PRISCUS**: >2 mg/d: "Risk of falling (muscle-relaxing effect) with risk of hip fracture; prolonged reaction times; psychiatric reactions (can also be paradoxical, e.g., agitation, irritability, hallucinations, psychosis); cognitive impairment; depression."

© 2024 Schietzel S. *JAMA Network Open.*

	**EU(7)**: >1 mg/d: “Risk of falling with hip fracture; prolonged reaction times; psychiatric reactions (can also be paradoxical, e.g. agitation, irritability, hallucinations, psychosis); cognitive impairment; depression.”
**Suggestion**	**Beers 2019**: Short and intermediate acting benzodiazepines: “Avoid; may be appropriate for seizure disorders, rapid eye movement sleep behavior disorder, benzodiazepine withdrawal, ethanol withdrawal, severe generalized anxiety disorder, and periprocedural anesthesia.” Delirium: “Avoid.” Dementia or cognitive impairment: “Avoid.” History of falls or fractures: “Avoid unless safer alternatives are not available. If the drug must be used, consider reducing use of other CNS-active medications that increase risk of falls and fractures (ie, antiepileptics, opioid-receptor agonists, antipsychotics, antidepressants, nonbenzodiazepine and benzodiazepine receptor agonist hypnotics, other sedatives/hypnotics) and implement other strategies to reduce fall risk.” Drug-drug interactions; any combination of three or more CNS-active drugs: “Avoid total of three or more CNS-active drugs; minimize number of CNS-active drugs.”<br>**Laroche**: “Dose of short- or intermediate- half-life benzodiazepine ≤ half the dose given in young subjects.”<br>**PRISCUS**: Alternatives: “Valerian; sedating antidepressants (trazodone, mianserin, mirtazapine); zolpidem (≤5 mg/d); opipramol; low-potency neuroleptic drugs (melperone, pipamperone); non-pharmacological treatment of sleep disturbances (sleep hygiene).” Precautions: “Clinical monitoring for adverse effects (cognitive function, vigilance, regular fall history, testing of gait steadiness, psychopathology, ataxia); dosing recommendation: lowest possible dose, up to half of the usual dose, taper in and out, shortest possible duration of treatment.”<br>**EU(7)**: Dose adjustment: “Reduce dose; use doses of 0.25–1 mg/d.” Alternatives: “Non-pharmacological treatment; low doses of short-acting benzodiazepines such as lormetazepam (≤0.5 mg/d), brotizolam (≤0.125 mg/d); antidepressants with anxiolytic profile (SSRI). If used as hypnotic or sedative consider: Mirtazapine; passiflora; low doses of short-acting benzodiazepines such as lormetazepam (≤0.5 mg/d), brotizolam (≤0.125 mg/d); zolpidem (≤5 mg/d), zopiclon (≤3.75 mg/d), zaleplon (≤5 mg/d); trazodone.”
**6. Quetiapine**	
**Why PIM**	**Beers 2019**: Antipsychotics, atypical: “Increased risk of cerebrovascular accident (stroke) and greater rate of cognitive decline and mortality in persons with dementia.” Delirium: “Avoid in older adults with or at high risk of delirium because of potential of inducing or worsening delirium.” Dementia or cognitive impairment: “Avoid because of adverse CNS effects. Antipsychotics are associated with greater risk of cerebrovascular accident (stroke) and mortality in persons with dementia.” History of falls or fractures: “May cause ataxia, impaired psychomotor function, syncope, additional falls.” Use with caution: “May exacerbate or cause SIADH or hyponatremia.” Drug-drug interactions; any combination of three or more CNS-active drugs: “Increased risk of falls.”<br>**Laroche**: Neuroleptics except olanzapine and risperidone: “Aggravation of cognitive impairment.”<br>**NORGEP**: Concomitant prescription of three or more drugs within the groups centrally acting analgesics, antipsychotics, antidepressants and/or benzodiazepines: “Increased risk of muscular weakness, falls, fractures and cognitive impairment.”
**Suggestion**	**Beers 2019**: Antipsychotics, atypical: “Avoid, except in schizophrenia or bipolar disorder, or for short-term use as antiemetic during chemotherapy. Avoid antipsychotics for behavioral problems of dementia and/or delirium unless nonpharmacological options (eg, behavioral interventions) have failed or are not possible and the older adult is threatening substantial harm to self or others.” Delirium: “Avoid.” Dementia or cognitive impairment: “Avoid.” History of falls or fractures: “Avoid unless safer alternatives are not available. If the drug must be used, consider reducing other CNS-active medications that increase risk of falls and fractures (ie, antiepileptics, antidepressants, nonbenzodiazepine benzodiazepine receptor agonist hypnotics, other sedatives/hypnotics) and implement other strategies to reduce fall risk.” Use with caution: “Monitor sodium level closely when starting or changing dosages in older adults.” Drug-drug interactions; any combination of three or more CNS-active drugs: “Avoid total of three or more CNS-active drugs; minimize number of CNS-active drugs.”<br>**Laroche**: No recommendation given.

© 2024 Schietzel S. *JAMA Network Open.*

	**NORGEP**: No recommendation given.
**7. Sodium picosulfate**	
**Why PIM**	**Laroche**: Stimulant laxatives: "Worsening of irritable bowel syndrome."<br>**EU(7)**: "Stimulant laxative. Adverse events include abdominal pain, fluid and electrolyte imbalance and hypoalbuminemia. May exacerbate bowel dysfunction."
**Suggestion**	**Laroche**: "Osmotic laxatives."<br>**EU(7)**: "Recommend proper dietary fibre and fluid intake; osmotically active laxatives: macrogol, lactulose."
**8. Ginkgo folium**	
**Why PIM**	**Laroche**: Cerebral vasodilators: "Questionable efficacy. No really proven efficacy while postural hypotension and fall risks are increased with most vasodilators."<br>**EU(7)**: Anti-dementia drugs: "No efficacy proven; increased risk of orthostatic hypotension and fall."
**Suggestion**	**Laroche**: "Therapeutic abstention."<br>**EU(7)**: "Non-pharmacological treatment; consider pharmacotherapy of Alzheimer-type dementia: acetylcholinesterase, memantine."
**9. Tramadol**	
**Why PIM**	**Beers 2019**: Use with caution: "May exacerbate or cause SIADH or hyponatremia." CrCl <30 ml/min: "CNS adverse effects."<br>**NORGEP**: Centrally acting analgesics: "Increased risk of muscular weakness, falls, fractures and cognitive impairment."<br>**Mann**: "Lowers seizure threshold, may lead to delirium, frequent unwanted side effects: Vomiting, vertigo, constipation."<br>**EU(7)**: Opioids: "More adverse effects in older people; CNS side effects such as confusion, vertigo and nausea."
**Suggestion**	**Beers 2019**: Use with caution: "Monitor sodium levels closely when starting or changing dosages in older adults." CrCl <30 ml/min: "Immediate release: reduce dose. Extended release: avoid."<br>**NORGEP**: No recommendations given.<br>**Mann**: "Paracetamol, metamizole, hydromorphone."<br>**EU(7)**: Sustained release: "Start low—go slow. Not to be used in cases of severe renal failure." Non-sustained release: "Start low—go slow; in persons older than 75 years, daily doses over 300 mg are not recommended. Start with 12.5 mg/8 h and progressive increases of 12.5 mg/8 h; maximum 100 mg/8 h. Reduce dose and extend the dosing interval for patients with severe renal failure." Alternative drugs: "Paracetamol; ibuprofen (≤3 × 400 mg/d or for a period shorter than one week); naproxen (≤2 × 250 mg/d or for a period shorter than one week). Opioids with lower risk of delirium (e.g. tilidine/naloxone, morphine, oxycodone, buprenorphine, hydromorphone)."
**10. Pregabalin**	
**Why PIM**	**Beers 2019**: Drug-drug interactions with opioids: "Increased risk of severe sedation-related adverse events, including respiratory depression and death." CrCl <60 ml/min: "CNS adverse effects."
**Suggestion**	**Beers 2019**: Drug-drug interactions with opioids: "Avoid; exceptions are when transitioning from opioid therapy to gabapentin or pregabalin, or when using gabapentinoids to reduce opioid dose, although caution should be used in all circumstances." CrCl <60 ml/min: "Reduce dose."
**11. Esomeprazole**	
**Why PIM**	Refer to pantoprazole.
**Suggestion**	Refer to pantoprazole.
**12. Loperamide**	

© 2024 Schietzel S. *JAMA Network Open.*

**Why PIM**	**EU(7)**: >2 days: "Risk of somnolence, constipation, nausea, abdominal pain and bloating. Rare adverse events include dizziness. May precipitate toxic megacolon in inflammatory bowel disease, may delay recovery in unrecognised gastroenteritis."
**Suggestion**	**EU(7)**: Dose adjustment: "Start with a dose of 4 mg followed by 2 mg in each deposition until normalisation of bowel; do not exceed 16 mg/d; use no longer than 2 days; may be useful in palliative care for persisting non-infectious diarrhoea." Alternatives: "Non-pharmacological measures, e.g. diet; phloroglucinol."
**13. Oxycodone (+ Naloxone)**	
**Why PIM**	**Beers 2019**: History of falls or fractures: "May cause ataxia, impaired psychomotor function, syncope, additional falls." Drug-drug interaction with benzodiazepines: "Increased risk of overdose." Drug-drug interaction with gabapentin, pregabalin: "Increased risk of severe sedation-related adverse events, including respiratory depression and death." Drug-drug interactions; any combination of three or more CNS-active drugs: "Increased risk of falls."<br>**NORGEP**: Centrally acting analgesics: "Increased risk of muscular weakness, falls, fractures and cognitive impairment."
**Suggestion**	**Beers 2019**: History of falls or fractures: "Avoid unless safer alternatives are not available. Avoid except for pain management in the setting of severe acute pain (eg, recent fractures or joint replacement). If the drug must be used, consider reducing use of other CNS-active medications that increase risk of falls and fractures (ie, antiepileptics, opioid-receptor agonists, antipsychotics, antidepressants, nonbenzodiazepine and benzodiazepine receptor agonist hypnotics, other sedatives/hypnotics) and implement other strategies to reduce fall risk." Drug-drug interaction with benzodiazepines: "Avoid." Drug-drug interaction with gabapentin, pregabalin: "Avoid; exceptions are when transitioning from opioid therapy to gabapentin or pregabalin, or when using gabapentinoids to reduce opioid dose, although caution should be used in all circumstances." Drug-drug interactions; any combination of three or more CNS-active drugs: "Avoid total of three or more CNS-active drugs; minimize number of CNS-active drugs."<br>**NORGEP**: No recommendations given.
**14. Chondroitin sulfate**	
**Why PIM**	**Laroche**: Concomitant use of 2 or more NSAIDs: "No enhancement of efficacy, albeit increase of adverse effect risk."<br>**NORGEP**: NSAID (or coxib) + ACE inhibitor (or ARB): "Increased risk of renal failure." NSAID + diuretic: "Reduced effect of diuretics." NSAID + glucocorticoid: "Increased risk of gastrointestinal bleeding. Risk of fluid retention." NSAID + SSRI: "Increased risk of gastrointestinal bleeding."
**Suggestion**	**Laroche**: "Use only one NSAID."<br>**NORGEP**: No recommendations given.
**15. Tramadol (+ Paracetamol)**	
**Why PIM**	Refer to tramadol.
**Suggestion**	Refer to tramadol.
**16. Nitrofurantoin**	
**Why PIM**	**Beers 2019**: "Potential for pulmonary toxicity, hepatotoxicity, and peripheral neuropathy, especially with long-term use; safer alternatives available."<br>**Laroche**: "Can induce renal insufficiency, pneumopathy, peripheral neuropathy, allergic reaction. Bacterial resistance in case of protracted use."<br>**PRISCUS**: "Unfavorable risk/benefit ratio, particularly with long-term use (pulmonary side effects, liver damage, etc.)"
**Suggestion**	**Beers 2019**: "Avoid in individuals with creatinine clearance < 30 ml/min or for long-term suppression."<br>**Laroche**: "Antibiotics with renal elimination according to the antibiogram."<br>**PRISCUS**: Alternatives: "Other antibiotics (e.g., cephalosporins, cotrimoxazole, trimethoprim—in accordance with sensitivity and resistance testing, as far as possible).

© 2024 Schietzel S. *JAMA Network Open.*

	Non-pharmacological measures: more fluid intake, incontinence aids." Precautions: "Monitoring of renal, pulmonary, and hepatic function."
**17. Naproxen + Esomeprazole**	
**Why PIM**	**Beers 2019**: For naproxen refer to ibuprofen; for esomeprazole refer to pantoprazole.<br>**Laroche**: For naproxen refer to ibuprofen.<br>**NORGEP**: For naproxen refer to ibuprofen.<br>**Mann**: For naproxen refer to ibuprofen.<br>**EU(7)**: For esomeprazole refer to pantoprazole.
**Suggestion**	**Beers 2019**: For naproxen refer to ibuprofen; for esomeprazole refer to pantoprazole.<br>**Laroche**: For naproxen refer to ibuprofen.<br>**NORGEP**: For naproxen refer to ibuprofen.<br>**Mann**: For naproxen refer to ibuprofen.<br>**EU(7)**: For esomeprazole refer to pantoprazole.
**18. Spironolcatone**	
**Why PIM**	**Beers 2019**: CrCl <30 ml/min: "Increased potassium."<br>**EU(7)**: >25 mg/d: "Higher risk of hyperkalaemia and hyponatremia in older people, especially if doses >25 mg/d, requiring periodic controls."
**Suggestion**	**Beers 2019**: CrCl <30 ml/min: "Avoid."<br>**EU(7)**: Dose adjustment: "Reduce dose in cases of moderate renal insufficiency. GFR ≥50 mL/min/1.73 m: initial dose 12.5–25 mg/d, increase up to 25 mg 1–2/d; GFR 30–49 mL/min/1.73 m: initial dose 12.5 mg/d, increase up to 12.5–25 mg/d; reduce dose if potassium levels increase or renal function worsens. GFR <10 mL/min: avoid." Alternatives: "Consider alternatives depending on the indication; exclude PIMs."
**19. Acemetacin**	
**Why PIM**	**Laroche**: Refer to ibuprofen.<br>**NORGEP**: Refer to ibuprofen.<br>**PRISCUS**: "Very high risk of gastrointestinal hemorrhage, ulceration, or perforation, which may be fatal."<br>**Mann**: Refer to ibuprofen.
**Suggestion**	**Laroche**: Refer to ibuprofen.<br>**NORGEP**: Refer to ibuprofen.<br>**PRISCUS**: Alternatives: "Paracetamol, (weak) opioids (tramadol, codeine), weak NSAID (e.g., ibuprofen)." Precautions: "Use in combination with protective agents, e.g., PPI; follow-up for gastrointestinal manifestations (gastritis, ulcer, hemorrhage); monitoring of renal function; monitoring of cardiovascular function (blood pressure, signs of congestive heart failure); dosing recommendation: shortest possible duration of therapy."<br>**Mann**: Refer to ibuprofen.
**20. (Metformin +) Sitagliptin**	
**Why PIM**	**EU(7)**: "Limited safety data available for adults aged ≥75 years old. Subjects aged 65 to 80 had higher plasma concentrations than younger subjects. Risk of hypoglycemia, dizziness, headache and peripheral oedema."
**Suggestion**	**EU(7)**: "Reduce dose to 50 mg/d in cases of renal failure (CrCl 30-50 mL/min); reduce dose to 25 mg/d in cases of severe renal insufficiency (CrCl <30 mL/min)."

© 2024 Schietzel S. *JAMA Network Open.*

References

1. Supplemental Material for: Potentially Inappropriate Medication Use in Primary Care in Switzerland. zenodo; 2024. https://doi.org/10.5281/zenodo.10572225. Accessed 2024/1.
2. By the 2019 American Geriatrics Society Beers Criteria® Update Expert Panel. American Geriatrics Society 2019 updated AGS Beers Criteria® for Potentially Inappropriate Medication Use in Older Adults. J Am Geriatr Soc. 2019;67(4):674-694.
3. Holt S, Schmiedl S, Thürmann PA. Potentially inappropriate medications in the elderly: the PRISCUS list. Dtsch Arztebl Int. 2010;107(31-32):543-551.
4. Laroche ML, Charmes JP, Merle L. Potentially inappropriate medications in the elderly: a French consensus panel list. Eur J Clin Pharmacol. 2007;63(8):725-731.
5. Mann E, Böhmdorfer B, Frühwald T, et al. Potentially inappropriate medication in geriatric patients: the Austrian consensus panel list. Wien Klin Wochenschr. 2012;124(5-6):160-169.
6. Renom-Guiteras A, Meyer G, Thürmann PA. The EU(7)-PIM list: a list of potentially inappropriate medications for older people consented by experts from seven European countries. Eur J Clin Pharmacol. 2015;71(7):861-875.
7. Rognstad S, Brekke M, Fetveit A, Spigset O, Wyller TB, Straand J. The Norwegian General Practice (NORGEP) criteria for assessing potentially inappropriate prescriptions to elderly patients. A modified Delphi study. Scand J Prim Health Care. 2009;27(3):153-159.

© 2024 Schietzel S. *JAMA Network Open.*
