# Supplementary material for: Potentially Inappropriate Medication Use in Primary Care in Switzerland
Source: JAMA Netw Open. 2024 Jun 21;7(6):e2417988. doi: 10.1001/jamanetworkopen.2024.17988 (PMC11193127; doi:10.1001/jamanetworkopen.2024.17988)
Supplement: Supplement 2. — Data Sharing Statement [file jamanetwopen-e2417988-s002.pdf]

Data Sharing Statement

Schietzel. Potentially Inappropriate Medication Use in Primary Care in Switzerland. *JAMA Netw Open*. Published June 21, 2024. doi:10.1001/jamanetworkopen.2024.17988

Data
Data available: Yes
Data types: Deidentified participant data
How to access data: Requests should be addressed to the corresponding author: thomas.grischott@usz.ch
When available: With publication

Supporting Documents
Document types: Statistical/analytic code
How to access documents: Definitions, code snippets, and additional results in electronic form are available from: Supplemental Material for: Potentially Inappropriate Medication Use in Primary Care in Switzerland. zenodo; 2024. https://doi.org/10.5281/zenodo.10572225.
When available: With publication

Additional Information
Who can access the data: Deidentified participant data may be shared for research collaborations.
Types of analyses: Any non-commercial research purposes.
Mechanisms of data availability: Collaboration proposals with data access requests must include an analysis plan and be approved by the FIRE research group prior to data release.
